# Neuropsychiatric symptoms associated with family caregiver burden and depression

**DOI:** 10.1590/1980-57642021dn15-010014

**Published:** 2021

**Authors:** Lais Lopes Delfino, Ricardo Shoiti Komatsu, Caroline Komatsu, Anita Liberalesso Neri, Meire Cachioni

**Affiliations:** 1 School of Medical Sciences, Universidade Estadual de Campinas – Campinas, SP, Brazil.; 2 Geriatrics and Gerontology Discipline, Faculdade de Medicina de Marília – Marília, SP, Brazil.; 3 Hospital São Paulo, Universidade Federal de São Paulo – São Paulo, SP, Brazil.; 4 School of Arts, Science, and Humanities, Universidade de São Paulo – São Paulo, SP, Brazil.

**Keywords:** behavioral symptoms, Alzheimer's disease, depression, caregivers, sintomas comportamentais, doença de Alzheimer, depressão, cuidadores

## Abstract

Alzheimer's disease (AD) is a progressive and degenerative condition affecting several cognitive areas, with a decline in functional abilities and behavioral changes.

**Objective:**

To investigate the association between neuropsychiatric symptoms in older adults with AD and caregiver burden and depression.

**Methods:**

A total of 134 family caregivers of older people diagnosed with AD answered a questionnaire with sociodemographic data and questions concerning the care context, neuropsychiatric symptoms, caregiver burden, and depressive symptoms.

**Results:**

Results revealed that 95% of older adults had at least one neuropsychiatric symptom, with the most common being: apathy, anxiety, and depression. Among the 12 neuropsychiatric symptoms investigated, 10 were significantly associated with caregiver burden, while 8 showed significant correlations with depressive symptoms.

**Conclusions:**

Neuropsychiatric symptoms were related to caregiver burden and depressive symptoms. In addition to the older adult with AD, the caregiver should receive care and guidance from the health team to continue performing quality work.

## RESUMO.

A doença de Alzheimer (DA) é progressiva e degenerativa, afetando diversas áreas cognitivas com declínio nas habilidades funcionais e alterações comportamentais.

**Objetivo:**

Investigar a associação entre presença de sintomas neuropsiquiátricos apresentados por idosos com doença de Alzheimer e sobrecarga, e depressão dos cuidadores.

**Métodos:**

Um total de 134 cuidadores familiares de idosos com diagnóstico da doença de Alzheimer responderam a um questionário com dados sociodemográficos e questões referentes ao contexto de cuidado, sintomas neuropsiquiátricos, sobrecarga e depressão do cuidador.

**Resultados:**

Os resultados revelaram que 95% dos idosos apresentaram pelo menos um sintoma neuropsiquiátrico. A apatia, a ansiedade e a depressão foram os sintomas neuropsiquiátricos mais frequentes nos idosos. Dos 12 sintomas neuropsiquiátricos investigados, 10 associaram-se significativamente à sobrecarga do cuidador (exceto ansiedade e alteração alimentar), e oito sintomas neuropsiquiátricos apresentaram correlações significativas com os sintomas de depressão.

**Conclusão:**

A presença de determinados sintomas neuropsiquiátricos está relacionada com a sobrecarga e com sintomas de depressão apresentados pelos cuidadores. Além do idoso com doença de Alzheimer, o cuidador deve receber cuidados e orientação da equipe de saúde para que possa continuar desempenhando sua função com qualidade.

## INTRODUCTION

Alzheimer's disease (AD) is a progressive and degenerative brain condition that affects multiple cognitive areas and results in a decline in functional abilities and behavioral changes.^1^ The literature widely recognizes that AD clinical manifestations are not limited to cognitive changes but also include neuropsychiatric symptoms (NPSs),^2^ that is, a heterogeneous group of perceptual, thought, mood, personality, and behavioral disturbances.^3^ ^,^ ^4^ The terms “neuropsychiatric symptoms” and “behavioral and psychological symptoms of dementia” are used interchangeably in the literature. According to population studies, more than 80% of AD patients develop behavioral and psychological symptoms at some point during the course of the disease.^3^ ^,^ ^5^

NPSs in patients with dementia are associated with worse prognosis, higher health care costs, greater impairment in daily functioning and quality of life, faster cognitive decline, early institutionalization, as well as increased mortality and caregiver burden.^6^ ^–^ ^8^ Multiple factors contribute to the manifestation of NPSs, including aspects related to the person with dementia, the pathophysiological process of the disease, acute conditions, unmet needs, and pre-existing personality factors. Environmental conditions, caregiver-related factors, neglected needs, patient and caregiver personality, among other variables, can also lead to the manifestation of NPSs.^9^ Stress and depression increase when a caregiver manages NPSs, and these symptoms can be triggered or exacerbated when a caregiver is stressed or depressed.^10^

The burden experienced by caregivers has many causes, such as the constant and increasing need to supervise the patient, the older adult's physical and cognitive dependence, the lack of support from other family members, family conflicts, financial difficulties, and social deprivation.^11^ ^,^ ^12^ Researchers have shown that NPSs of an older adult affected by AD are some of the main determinants of caregiver burden.^11^ ^,^ ^13^ NPSs are reported as more stressful for caregivers than cognitive and functional problems, perhaps due to the unstable nature of these symptoms. While the functional and cognitive trajectories of the patient with dementia follow a constant and expected decline, behavioral problems may fluctuate, which may leave the caregiver less prepared to deal with them properly. In addition, NPSs alter the patient's personality and may be more dramatic reminders of the major changes undergone by the patient and the loss experienced by the caregiver.^13^ ^,^ ^14^

The results of a Brazilian population study involving a sample of 10,853 individuals, including 205 caregivers, showed that caregivers of people with AD presented a substantially higher risk of depressive symptoms, major depressive disorder, anxiety, insomnia, hypertension, pain, and diabetes (all with p<0.015).^15^ These negative outcomes require the development of new strategies for prevention, early detection, and interventions to deal with dementia caregiver burden.

Many studies that provided evidence of the association between NPSs and caregiver burden and depression investigated the variables globally; thus, the effect of each symptom on caregiver burden and depression needs to be further explored in the literature.^13^ ^,^ ^16^ Our research hypothesized that NPSs are associated with caregiver burden and/or depressive symptoms. Knowledge of the impact that each NPS has on the caregiver's life contributes to identifying those at high risk of stress so that health services can be tailored to the needs of these patients, and admission to long-term care facilities can be delayed. This study aimed to investigate the relationship between each NPS presented by people with AD and the caregiver burden and depressive symptoms.

## METHODS

### Participants

The study protocol was approved by the Ethics Committee of the Universidade Estadual de Campinas (UNICAMP) (CAAE 47901615.5.0000.5404). The sample comprised 134 caregivers of patients with AD recruited from a geriatric clinic in Marília, São Paulo, Brazil, using a non-probabilistic convenience sample. All subjects provided written informed consent for participation in accordance with the study protocol.

The inclusion criteria were: being a primary caregiver, that is, providing daily care in routine activities for at least 4 hours a day, being the caregiver of an older adult diagnosed with AD, according to the criteria recommended by the National Institute of Neurological and Communicative Disorders and Stroke/Alzheimer's Disease and Related Disorders Association (NINCDS-ADRDA).^17^

After the screening, all participants were assessed for the following exclusion criteria:

- caregivers of people with other diagnoses, such as cancer and psychiatric disorders (schizophrenia, bipolar disorder, obsessive-compulsive disorder, and others);
- caregivers of individuals with a score above the cut-off point on the Mini-Mental State Examination, based on the score suggested by Brucki et al.^1^ ^8^ (1 to 4 years of schooling: 22; 5 to 8 years: 24; over 9 years: 26);
- taking care of people living in nursing homes or those who are in a terminal stage according to medical evaluation.

### Interview procedures

First, medical records of the individuals diagnosed with AD were checked to collect information about the caregivers and the results of the Mini-Mental State Examination. Caregivers who met the criteria established in this study were contacted by telephone to schedule the interview. The interviews were conducted by a researcher trained to administer the selected instruments. The caregiver could not be accompanied by the patient during the interview, so the patient stayed in a waiting room, where they were monitored by the clinic staff. The duration of each interview ranged from 35 to 80 minutes. The mean interview length was 46 minutes.

### Measures

A questionnaire with items about sociodemographic aspects (age, education, income, occupation) and the relationship between caregiver and dementia care recipients (family care, co-residence, care time) was administered to the caregivers.

The participants answered the Neuropsychiatric Inventory (NPI).^19^ This questionnaire independently evaluates 12 behavioral domains (delusions, hallucinations, dysphoria/depression, anxiety, agitation/aggression, euphoria, disinhibition, irritability/emotional lability, apathy, aberrant motor activity, sleep and nighttime behavior change, and appetite and eating change). The caregiver initially responds to a screening question, and, in case of a positive result, the frequency and intensity of each item are evaluated. The total score for each domain is calculated by the equation frequency × severity. The total NPI score ranged from 0 to 144.

Furthermore, an additional scale, NPI Caregiver Distress (NPI-D), was developed and validated to provide a quantitative measure of the distress experienced by caregivers for each NPI symptom presented by the patient. Caregivers were asked to rate their emotional or psychological distress on a 6-point scale: 0 (not at all distressed), 1 (minimally distressed), 2 (mildly distressed), 3 (moderately distressed), 4 (severely distressed), and 5 (very severely or extremely distressed). The Brazilian versions of the NPI and NPI-D subscale were validated in 2008.^20^

Caregivers responded to Zarit Burden Interview (ZBI) to investigate burden.^21^ This scale consists of 22 questions with answers ranging from zero (never) to four (nearly always), reflecting the perception of the caregiver as to health, personal and social life, financial situation, personal well-being, and interpersonal relationships. Its score ranges from 0 to 88 and reveals the level of caregiver burden — the higher the score, the greater the perceived burden. ZBI was validated in Brazil with a sample of caregivers of people with psychiatric illnesses by Scazufca.^21^ In this study, the participants’ total scores were divided into: 0–23 (low burden), 24–26 (moderate burden), and ≥27 (high burden).

Caregivers also answered the Beck Depression Inventory.^22^ The original scale consists of 21 items, including symptoms and attitudes. The items refer to mood, pessimism, sense of failure, lack of satisfaction, guilt feelings, sense of punishment, self-dislike, self-accusation, suicidal wishes, crying, irritability, social withdrawal, indecisiveness, distortion of body image, work inhibition, sleep disturbance, fatigability, loss of appetite, weight loss, somatic preoccupation, and loss of libido. The score for each category ranges from zero to three, with zero meaning the absence of depressive symptoms and three representing the most intense ones. Thus, the minimum score is 0, the maximum is 63, and the sum of the scores of individual items provides a total score, which corresponds to the intensity of depression, classified as minimal, mild, moderate, or severe. The cut-off points adopted were those suggested by Kendall et al.:^23^ scores up to 15 for the subgroup “without depression”; 16 to 20 for the subgroup “dysphoria or mild depression”; 21 to 29 for the subgroup “moderate depression”; and 30 or more for “severe depression”.

### Data analysis

The sample profile was described through frequency tables of categorical variables, with absolute (n) and percentage (%) values, and descriptive statistics of numerical variables, expressed as mean and standard deviation. Chi-square and Fisher's exact tests were used to compare the categorical variables. The Mann-Whitney test was adopted to compare the groups with and without NPSs, and the Spearman's rank correlation test was used to investigate the correlations between variables. The significance level set for the statistical tests was 5%, that is, p<0.05. The analyses were performed in Statistical Social for the Social Sciences (SPSS), version 22 (IBM SPSS Statistics).

## RESULTS

Table 1 shows sociodemographic data, the frequency of caregiver burden and depressive symptoms, and the characteristics of people with AD. The results revealed a predominance of female caregivers, who co-reside with the family member, are the patient's adult children, present high burden, and do not have depressive symptoms. Most patients are women, use psychotropic medications, and have at least one NPS.

**Table 1:** Characterization of the sample of caregivers and people with Alzheimer's disease according to the variables investigated.

		Mean (SD) or frequency (%)
Caregiver		
	Age	58.24 (12.6)
	Gender (female)	107 (80)
	Schooling (years)	14 (3.9)
Hours spent caring		
	5 to 10 hours	64 (47)
	11 to 15 hours	7 (5)
	>16 hours	63 (47)
Lives with patient		
	Yes	78 (58)
	No	56 (42)
Work in the profession		
	Yes	86 (64)
	No	48 (36)
Income		
	1 to 5 MW	16 (12)
	3.5 to 5 MW	37 (28)
	>5 MW	81 (60)
Relationship		
	Son/daughter	86 (64)
	Husband/wife	34 (25)
	Brother/sister	5 (4)
	Other relatives	9 (7)
	Burden (ZBI total)	31.46 (10.3)
Burden (ZBI scores)		
	≤23	36 (27)
	24 to 26	17 (13)
	≥27	81 (60)
	Distress (NPI-D)	13 (9.07)
	Depressive symptoms (BDI)	6.26 (5.98)
Depressive symptoms (BDI scores)		
	0 to 15	122 (91)
	16 to 20	7 (5)
	21 to 29	5 (4)
Patient		
	Age	80 (7.9)
	Gender (female)	82 (61)
	Schooling (years)	8 (5.7)
	MMSE	18 (5.9)
Use of psychotropic medication		
	Yes	120 (90)
	No	14 (10)
	Diagnosis time (years)	3.3 (3.7)
Neuropsychiatric symptoms (NPI)		
	Yes	127 (95)
	No	7 (5)

SD: standard deviation; MW: minimum wage; ZBI: Zarit Burden Interview; NPI-D: Neuropsychiatric Inventory Caregiver Distress Scale; BDI: Beck Depression Inventory; MMSE: Mini-Mental State Examination.

**Figure 1:**
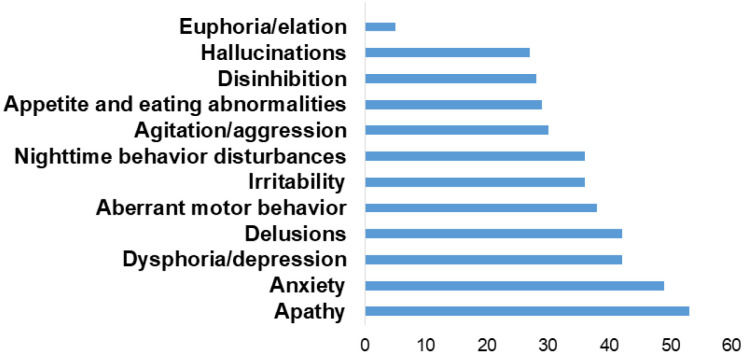
Frequency of patients with neuropsychiatric symptoms (%).

Figure 1 illustrates the frequency of patients with each NPS. Apathy, followed by anxiety, depression, and delusions were the most common symptoms among dementia care recipients and the more distressing, according to the caregiver (Table 2).

**Table 2:** Frequency of patients with neuropsychiatric manifestation and mean Neuropsychiatric Inventory score and distress reported by caregivers for each symptom.

	n (%)	Mean NPI (SD)	Mean distress (SD)
Apathy	71 (53)	3.40 (3.89)	1.61 (1.63)
Anxiety	65 (49)	2.76 (3.44)	1.40 (1.55)
Dysphoria/depression	57 (42)	2.12 (3.26)	1.21 (1.54)
Delusions	55 (42)	2.10 (3.26)	1.19 (1.56)
Aberrant motor behavior	51 (38)	2.87 (4.40)	1.13 (1.60)
Irritability	49 (36)	2.16 (3.38)	1.18 (1.66)
Nighttime behavior disturbances	49 (36)	2.64 (4.08)	1.12 (1.62)
Agitation/aggression	40 (30)	1.96 (3.64)	0.91 (1.53)
Appetite and eating change	39 (29)	2.20 (3.90)	0.93 (1.52)
Disinhibition	38 (28)	2.10 (3.79)	0.90 (1.49)
Hallucinations	37 (27)	1.37 (2.94)	0.79 (1.41)
Euphoria/elation	6 (5)	0.13 (0.88)	0.04 (0.36)

NPI: Neuropsychiatric Inventory; SD: standard deviation.

Significant differences were found between groups that presented or not each NPS (except anxiety and eating disorders) when compared to the burden scale scores. Regarding the Depression Inventory scores, the same group comparison demonstrated that only the mean scores of symptoms of anxiety, disinhibition, irritability, and eating change did not present statistically significant differences (Table 3). The results showed a greater burden and more depressive symptoms among caregivers in all NPSs investigated.

**Table 3:** Mean values and standard deviation of burden and depression for each neuropsychiatric symptom of the Neuropsychiatric Inventory.

		Burden (ZBI)			Depression (BDI)		
		Mean	SD	p-value	Mean	SD	p-value
Delusions	No	29.14	10.12	0.001	4.77	4.73	0.001
	Yes	34.78	9.9		8.4	6.91	
Hallucinations	No	29.63	9.91	<0.001	5.32	5.36	0.004
	Yes	36.24	10.16		8.73	6.84	
Agitation/aggression	No	29.04	9.27	<0.001	5.33	5.5	0.003
	Yes	37.13	10.73		8.45	6.53	
Dysphoria/depression	No	28.74	9.36	<0.001	4.96	5.05	0.004
	Yes	35.12	10.62		8.02	6.69	
Anxiety	No	30.74	9.42	0.558	6.12	6.26	0.496
	Yes	32.22	11.32		6.42	5.71	
Euphoria/elation	No	30.93	10.04	0.003	5.99	5.76	0.016
	Yes	48.5	5.97		15	7.07	
Apathy	No	28.89	10.66	0.004	4.98	5.88	0.004
	Yes	33.73	9.62		7.39	5.88	
Disinhibition	No	30.06	9.97	0.043	6.07	5.93	0.422
	Yes	34.97	10.67		6.74	6.16	
Irritability/lability	No	28.81	9.02	<0.001	5.74	5.76	0.174
	Yes	36.04	11.04		7.16	6.3	
Aberrant motor behavior	No	30.12	10.65	0.041	5.41	5.4	0.031
	Yes	33.63	9.62		7.65	6.64	
Nighttime behavior disturbances	No	29.4	9.79	0.003	5.48	5.84	0.01
	Yes	35.02	10.48		7.61	6.02	
Appetite and eating change	No	30.68	9.49	0.203	6.05	6.02	0.434
	Yes	33.33	12.19		6.77	5.91	

p-value for the Mann-Whitney test to compare values between the two groups (those who presented and did not present each NPS). ZBI: Zarit Burden Interview; BDI: Beck Depression Inventory; SD: standard deviation; NPS: neuropsychiatric symptom.

When analyzing the correlations between each NPS and the total burden and depression scores, the findings indicated that the studied variables are positively associated. Namely, the greater the presence of NPSs, the greater the burden and depression scores. Table 4 presents the statistically significant correlations.

**Table 4:** Correlations between neuropsychiatric symptoms of people with Alzheimer's disease and caregiver burden and depression.

	Burden (ZBI)		Depression (BDI)	
	r	p-value	r	p-value
Delusions	0.32	0.000	0.28	0.001
Hallucinations	0.31	0.000	0.23	0.008
Agitation/aggression	0.37	<0.0001	0.25	0.003
Dysphoria/depression	0.35	<0.0001	0.22	0.010
Anxiety	0.12	0.231	0.05	0.593
Euphoria/elation	0.26	0.003	0.21	0.016
Apathy	0.23	0.008	0.18	0.04
Disinhibition	0.21	0.016	0.09	0.304
Irritability	0.33	0.000	0.12	0.167
Aberrant motor behavior	0.17	0.048	0.17	0.054
Nighttime behavior disturbances	0.24	0.005	0.23	0.008
Appetite and eating change	0.11	0.211	0.06	0.459
NPSs (total)	0.44	<0.0001	0.32	0.000

ZBI: Zarit Burden Interview; BDI: Beck Depression Inventory; r: Spearman's rank correlation coefficient; NPSs: neuropsychiatric symptoms.

## DISCUSSION

This study investigated the relationship between each NPS in people with AD and caregiver burden and depression. The results showed a high prevalence of patients diagnosed with AD who had at least one NPS (95%). Apathy (53%), anxiety (49%), and depression (42%) were the most frequent symptoms and also the ones that caused greater distress for the caregiver, according to the NPI-D score.

Our findings on the frequency of people with dementia who presented NPSs are in agreement with studies elaborated in Brazil and other countries. Tiel et al.^24^ investigated NPSs in a sample of older Brazilians diagnosed with AD and found that 90.8% of the sample had one or more symptoms, among which psychomotor agitation, aberrant motor behavior, and apathy were the most prevalent. In the population study conducted by Siafarikas et al.,^4^ 91% of people with dementia presented at least one NPS, with the most frequent being agitation, apathy, and nocturnal behavior. A meta-analysis of studies on the prevalence of NPSs in AD patients, dating from 1964 to 2014, revealed that the most frequent NPS was apathy, with an overall prevalence of 49%, followed by depression, aggression, anxiety, and sleep disorder. The least common NPS was euphoria, with a total prevalence of 7%.^25^ Data from studies on the most common NPS manifestation in AD present discrepancies. However, apathy appears to be one of the most frequent NPSs in people with AD.^5^

Apathy comprises a spectrum of symptoms that includes lack of initiative, interest, motivation, energy, and enthusiasm to start some activity compared to the previous level of functioning of the patient and that is in disagreement with their age or culture.^26^ According to Sherman et al.,^27^ apathetic patients require more support, management, and resource utilization, therefore, generating high levels of attrition for caregivers. The distress of the caregiver of a patient with apathy can also be explained by the greater disability that this NPS imposes on the patients and by the feeling of frustration in the caregivers. The lack of motivation and interest in performing activities compromise the rehabilitation of these patients.^28^ Anxiety and depression were also the NPSs that increased distress, as reported by caregivers. In the study by Liu et al.,^26^ patient depression was highly associated with caregiver burden.

Our data revealed that most caregivers of dementia care recipients (60%) participating in this study presented high burden. The mean ZBI score was 31.47, similar to that found in other studies.^6^ ^,^ ^29^ This result has been discussed in the national and international literature. Caregivers of AD patients suffer more than those of physically frail older people, given the specific symptoms experienced by dementia patients, such as behavioral problems, disorientation, personality change, need for continuous supervision, as well as the caregiver isolation due to the patient's behavioral problems and the progressive deterioration of the patient's condition, which reduces or eliminates a long-term prospect of improvement, contributing to increased caregiver burden.^30^

Caregivers also feel more burdened when they have to deal with NPSs. This fact was confirmed by the data correlations between mean burden scores and NPSs, which revealed that the higher the caregiver burden, the greater the number of NPSs in dementia care recipients. These results corroborate other studies that identified a positive association between burden and NPSs.^6^ ^,^ ^30^

Only anxiety and appetite change were not significantly associated with burden. Although anxiety was one of the symptoms considered to be more stressful by the caregiver, the comparison test between groups with and without NPSs and the correlation test showed that anxiety was not significantly related to burden. This finding indicates that caregiver burden does not necessarily depend on the frequency or severity of the NPS presented. Similar results were reported by Huang et al.^31^

Concerning appetite change, a systematic review conducted by Terum et al.^23^ showed that this symptom had the weakest statistical association with caregiver burden. In this review, irritability, followed by agitation/aggression, delusions, and apathy were the symptoms that contributed to a greater caregiver burden.

A study of 881 caregivers aiming to investigate the factors associated with caregiver burden according to different degrees of cognitive impairment in AD patients revealed that aggressiveness, agitation, aberrant motor behavior, apathy, and sleep disorders were strongly associated with caregiver burden in the early and moderate stages of AD.^8^

Aggression can be the sole determinant of greater caregiver burden and early institutionalization.^32^ Aberrant motor behavior and nighttime change can increase the burden because patients need attention and constant supervision, which, in turn, can cause a more stressful situation for caregivers. Patients who experience changes in the sleep-wake cycle may have more NPSs, such as agitation, irritability, and apathy, resulting in high levels of caregiver burden.^8^

In this study, caregivers presented a low score of depressive symptoms evaluated by the Beck Depression Inventory. This result contrasts with data from other surveys, in which symptoms of depression are common in caregivers of patients with AD.^26^ One possible explanation is that most caregivers in this study are the patient's adult children (64%). According to a meta-analysis performed by Pinquart and Sorensen,^33^ adult children caregivers have lower levels of depressive symptoms than spouse caregivers. Adult children caregivers report fewer health problems and spend less time on care tasks compared to spouse caregivers. Although caregivers did not present high scores of depressive symptoms, when investigating depression in the presence of each NPS, the mean depression score increased for all NPSs investigated.

A systematic review of articles published between 1980 and 2015 investigated the role of individual NPSs as to their impact on different measures of the family caregiver well-being, revealing that depressive behaviors were the most distressing for them, followed by agitation/aggression and apathy.^16^ In another systematic review, patient depression was the symptom most often associated with caregiver depression. The three most commonly reported impactful symptoms were: patient depression affecting caregivers in 40% of studies, aggression in 50%, and sleep disturbances in 43%.^34^

Offering support to caregivers in coping and managing NPSs of older adults with AD is crucial. In Brazil, the great difficulty in recognizing symptoms as part of a dementia process is well known. Many still consider NPSs a result of the aging process and lack information on how to deal with the patient's dysfunctional behavior. Therefore, before providing information to those involved, the relationship between the caregiver and the older person with dementia must be understood from the caregiver's behavioral and emotional point of view. Certainly, it is possible to offer tools for the caregiver, with resources that impact the quality of life of both those who receive and provide care.

Many AD patients (95%) had at least one NPS. The caregivers of this sample had high levels of burden and low depression scores. Due to the high rate of caregiver burden and the strong association with NPSs, health professionals, especially physicians and gerontologists, should pay close attention to the burden of caregivers of people with AD. The results of this study represent an important reference material for clinicians to manage NPSs hierarchically. Agitation, depression, and delusions were the three main symptoms significantly associated with depression and caregiver burden. Therefore, the successful management of these symptoms is clinically important, especially to reduce caregiver depression and burden.

This study contributes to understanding some caregivers’ characteristics associated with AD patients, such as knowledge, and can be useful in individualizing educational objectives for caregivers. These caregivers’ behavioral and emotional characteristics should be considered primary endpoints in the overall care of AD patients.
